# Supplementary material for: Inhibition of In Vitro Infection of Hepatitis B Virus by Human Breastmilk
Source: Nutrients. 2022 Apr 8;14(8):1561. doi: 10.3390/nu14081561 (PMC9031155; doi:10.3390/nu14081561)
Supplement: Supplementary file 1 [file nutrients-14-01561-s001.zip › nutrients-1627130-supplementary.pdf]

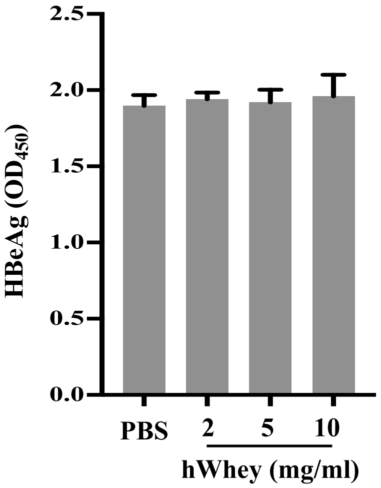

Supplementary Figure S1: Human whey does not bind to anti-HBs. Anti-HBs coated microplate wells were incubated with PBS (controls) or human whey (hWhey) at room temperature for 30 minutes. After wash, the plate was used to detect recombinant HBsAg using ELISA. Data are presented as means ± SD.

A

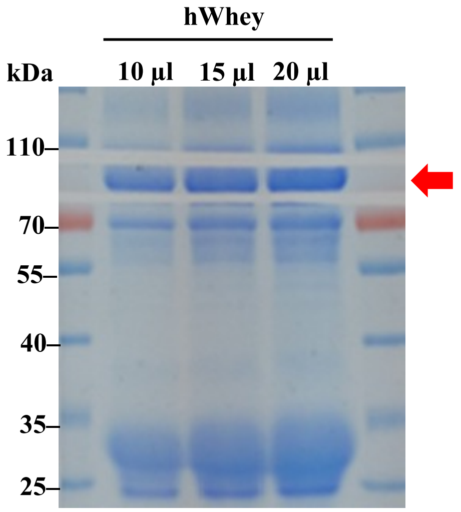
hWhey
kDa
10 µl
15 µl
20 µl
110
70
55
40
35
25

B

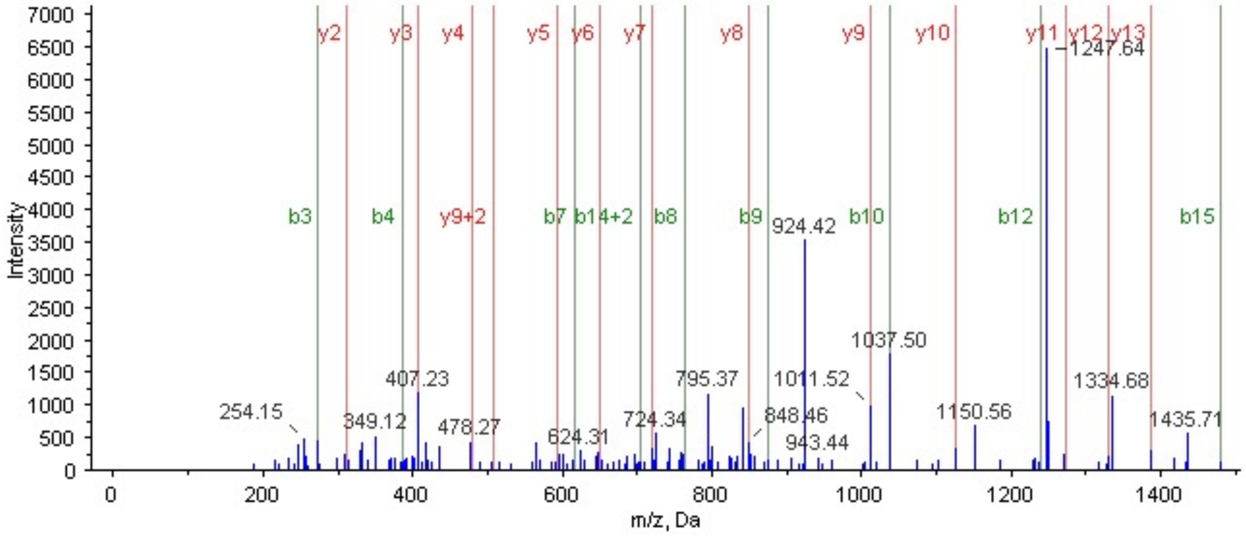
Intensity
m/z, Da
y2
y3
y4
y5
y6
y7
y8
y9
y10
y11
y12
y13
b3
b4
y9+2
b7
b14+2
b8
b9
b10
b12
b15
1247.64
924.42
1037.50
407.23
795.37
1011.52
1334.68
254.15
349.12
478.27
724.34
848.46
1150.56
1435.71
624.31
943.44

C

Con...	Conf	Sequence	Modifications	Cleavages	ΔMass	Prec MW	z	Sc	Spectrum	Type
2.00	99	ADAVTLDGGFIYEAGLAPYK	Dehydrated(D)@7		-0.0027	2052.01...	2	19	1.1.1.1277.26	Winner
2.00	99	ADFALLCLDGK	Carbamidomethyl(C) @7	cleaved L-...	-0.0011	1221.60...	2	19	1.1.1.1244.11	Winner
2.00	99	AFSSQEPYFSYSGAFK		cleaved C-...	-0.0033	1814.81...	2	21	1.1.1.1213.24	Winner
2.00	99	AVTLDGGFIYEAGLAPYK		cleaved D-...	-0.0018	1883.96...	2	19	1.1.1.1277.25	Winner
2.00	99	CAFSSQEPYF	Carbamidomethyl(C) @1	cleaved F-...	-0.0047	1234.49...	2	14	1.1.1.1226.9	Winner
2.00	99	CAFSSQEPYFSYSGA	Carbamidomethyl(C) @1	cleaved A-...	-0.0068	1699.67...	2	18	1.1.1.1226.27	Winner
2.00	99	CAFSSQEPYFSYSGAFK	Carbamidomethyl(C) @1 Dehydrated(S)@5 Deamidated(Q)@6		0.0031	1957.82...	2	23	1.1.1.1282.26	Winner
2.00	99	CAFSSQEPYFSYSGAFKCLR	Carbamidomethyl(C) @1 Carbamidomethyl(C) @18	missed K-C...	0.0062	2404.06...	3	14	1.1.1.1221.23	Winner
2.00	99	CGLVPVLAENY	Carbamidomethyl(C)@1	cleaved Y-K...	-0.0054	1233.60...	2	18	1.1.1.1273.4	Winner
2.00	99	CGLVPVLAENYK	Carbamidomethyl(C) @1		0.0054	1361.70...	2	20	1.1.1.1183.11	Winner
2.00	99	CLAENAGDVAFVK	Carbamidomethyl(C) @1		-0.0014	1392.66...	2	21	1.1.1.1149.20	Winner
2.00	99	CLAENAGDVAFVKDVTVLQNT...	Carbamidomethyl(C) @1 Carbamidomethyl(K) @13	missed K-D...	0.0269	3419.63...	3	20	1.1.1.1241.31	Winner
2.00	99	CLRDGAGDVAFIR	Carbamidomethyl(C) @1 Deamidated(R)@3 Dehydrated(D)@4	missed R-D...	-0.0031	1431.68...	2	20	1.1.1.1223.14	Winner

D

MKLVFLVLLFLGALGLCLAGRRRSVQWCAVSQPEATKCFQWQRNMRKVRGPPVSCIKRDSPIQCIQAIAENRADAVTLDGGFIYEA
GLAPYKLRPVAAEVYGTERQPRTHYYAVAVVKKGGSFQLNELQGLKSCHTGLRRTAGWNVPIGTLRPFLNWTGPPEPIEAAVARFF
SASCVPGADKGQFPNLCRLCAGTGENKCAFSSQEPYFSYSGAFKCLRDGAGDVAFIRESTVFEDLSDEAERDEYELLCPDNTRKPV
DKFKDCHLARVPSHAVVARSVNGKEDAIWNLLRQAQEKFGKDKSPKFQLFGSPSGQKDLLFKDSAIGFSRVPPRIDSGLYLGSGYF
TAIQNLRKSEEEVAARRARVVWCAVGEQELRKCNQWSGLSEGSVTCSSASTTEDCIALVLKGEADAMSLDGGYVYTAGKCGLVPVL
AENYKSQQSSDPDPNCVDRPVEGYLAVAVVRRSDTSLTWNSVKGKKSCHTAVDRTAGWNIPMGLLFNQTGSCKFDEYFSQSCAPGS
DPRSNLCALCIGDEQGENKCVPNSNERYYGYTGAFRCLAENAGDVAFVKDVTVLQNTDGNNNEAWAKDLKLADFALLCLDGKRKPV
TEARSCHLAMAPNHAVVSRMDKVERLKQVLLHQQAKFGRNGSDCPDKFCLFQSETKNLLFNDNTECLARLHGKTTYEKYLGPQYVA
GITNLKKCSTSPLLEACEFLRK

Supplementary Figure S2: Identification of human lactoferrin as HBsAg-binding protein in human whey by mass spectrometry analysis. (A) Human whey proteins were separated in SDS-PAGE, and the gel was cut at 80 kD (indicated by the red arrow) for mass spectrometry analysis. (B) The obtained mass-to-charge ratio
(m/z) mass spectrum. (C) The total number of identified peptide sequences (peptide spectrum matches, PSMs) for human lactoferrin. (D) The identified peptide sequences were with 93.4% amino acid residues identical with the human lactoferrin. Green: identified PSMs.

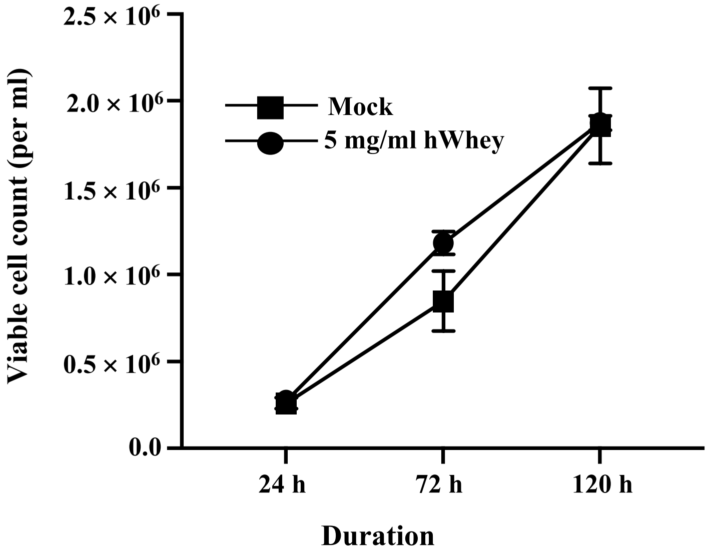

Supplementary Figure S3: Human whey at 5 mg/ml does not show any cytotoxic effect on HepG2-NTCP cells. HepG2-NTCP cells were incubated with 5 mg/ml human whey (hWhey) or PBS for 24 hours. The culture medium was replaced with fresh medium after the incubation, and the cells were kept for up to 120 hours. Cell numbers were counted at the indicated time point after incubation. Data are presented as means ± SD.
